# The Sparrow (Modified Lazy-S) Incision: A Practical Alternative to Level 1 Oncoplastic Techniques in Breast-Conserving Surgery

**DOI:** 10.3390/jcm14217706

**Published:** 2025-10-30

**Authors:** Berrin Papila, Mehmet Velidedeoglu

**Affiliations:** Department of General Surgery, Cerrahpasa Faculty of Medicine, Istanbul University-Cerrahpasa, 34098 Istanbul, Turkey; mehmet.dedeoglu@iuc.edu.tr

**Keywords:** breast neoplasms, body image, breast-conserving surgery, treatment

## Abstract

**Background:** The Sparrow (Modified Lazy-S) incision was developed as an alternative to the level 1 oncoplastic approach in breast-conserving surgery. Unlike the standard Lazy-S incision, both ends are more curved, a central skin islet is preserved, and the incision can be safely applied in all four breast quadrants, either horizontally or vertically. Large anterior skin islets facilitate the safe excision of tumors located near the skin, thereby reducing the risk of positive anterior margins. This technique is straightforward and does not require specialized training. **Methods:** Patients with invasive carcinoma who underwent breast-conserving surgery using the Sparrow incision at Istanbul University-Cerrahpasa Breast Clinic between January 2017 and January 2020 were retrospectively screened. **Results:** All 57 patients underwent the Sparrow incision. The overall complication rate was 8.8%, consistent with previously reported rates (9.8–10.97%). Patient-reported outcomes, assessed using Breast-Q™ modules, demonstrated high levels of satisfaction in terms of breast shape, symmetry, and psychosocial well-being. **Conclusions:** The Sparrow incision is an oncologically safe technique with potential advantages, as it does not prolong operative time or require additional instruments. It permits excision of previous biopsy sites and can be applied in patients undergoing neoadjuvant therapy or presenting with multifocal tumors. Patient-reported outcomes suggest favorable esthetic and psychosocial results.

## 1. Introduction

Breast cancer is the most common cancer in women [1], and its diagnosis and treatment represent a significant physical and emotional challenge. Like the disease itself, changes in the physical appearance of the breast after surgery can disrupt psychological well-being, body image, and overall quality of life [2,3,4]. Tumor location within the breast further influences surgical planning, cosmetic outcomes, and patient satisfaction. Breast cancers are most frequently located in the upper outer quadrant (45%), followed by the upper inner quadrant (15%), lower outer quadrant (10%), and lower inner quadrant (5%) [5]. Breast-conserving surgery (BCS) aims to remove the tumor with clear oncological margins while preserving as much healthy breast tissue as possible, and it has become a widely accepted standard due to its ability to maintain breast esthetics without compromising oncological safety [6].

Over the years, there has been a continuous search for alternatives to classical incisions in breast-conserving surgery, motivated by the need to optimize both oncological outcomes and cosmetic results. Tan et al. introduced the Boomerang incision in 2007 [7] and subsequently modified it in 2015 [8], offering better adaptability for tumors in specific quadrants. Nevertheless, these incisions often had limitations in terms of breast size, tumor location, and closure complexity, and no single incision was available that could be safely applied to all quadrants and breast volumes.

To overcome these challenges, we developed the Sparrow (Modified Lazy-S) incision, which incorporates a curved design with a central skin islet, allowing for precise tumor excision with safe margins while minimizing distortion of the breast contour. This incision is versatile, applicable in all four quadrants, and particularly advantageous for tumors located near the skin, where anterior margin positivity is a concern. Furthermore, the Sparrow incision is straightforward to perform, does not require specialized training, and can be safely applied in small- and medium-volume breasts, making it a practical technique for general surgeons and in settings with limited oncoplastic support [9,10,11,12].

This study aimed to present our experience with the Sparrow (Modified Lazy-S) incision in breast-conserving surgery, evaluating its oncological safety, cosmetic outcomes, complication rates, and patient satisfaction. Additionally, we aimed to assess whether this technique could provide a simple, versatile, and reproducible alternative to classical incisions for level 1 oncoplastic procedures.

## 2. Materials and Methods

This study was conducted according to the guidelines of the Declaration of Helsinki and approved by the Istanbul University-Cerrahpasa, Cerrahpasa Medical Faculty Clinical Research Ethics Committee (number of approval: 56390; Date: 22 April 2020). Informed consents were obtained from all patients.

A total of 57 patients who underwent the Sparrow incision were included in the study. Patients who were admitted to the Istanbul University-Cerrahpaşa, Cerrahpaşa Medical Faculty Breast Clinic with a diagnosis of invasive carcinoma and decided to have breast-conserving surgery, who are candidates for resection of less than 20% percent of the breast tissue (level 1 oncoplastic surgery) at the multidisciplinary breast council, were screened between January 2017 and January 2020.

The patient’s age, tumor localization, tumor size, complications, and comorbidity data were retrospectively analyzed from the surgical, epicrisis, and pathology reports. In all cases, intraoperative pathological evaluation of the surgical margins was performed routinely. To evaluate the new incision, the BREAST-Q™ Breast Cancer Core Scale and the BCT Module Version 2.0 Psychosocial Well-Being and Satisfaction with Breasts modules were administered [13].

The Sparrow (Modified Lazy-S) incision was applied consecutively to all eligible patients who met the inclusion criteria during the study period. No patients were selectively excluded based on breast volume or degree of ptosis, as the technique was designed to be suitable for both small-volume and ptotic breasts.

### 2.1. Inclusion and Exclusion Criteria

The women who had multifocal tumors that were more than 4 cm away or had multicentric tumors, women for whom radiotherapy is contraindicated, centrally localized tumors in which the nipple areola complex is invaded, and women for whom breast conserving therapy is contraindicated are excluded from the study. Women who are candidates for level 1 oncoplastic surgery (less than 20% of breast tissue is removed) are included in the study.

### 2.2. Surgical Technique

**Figure 1:**
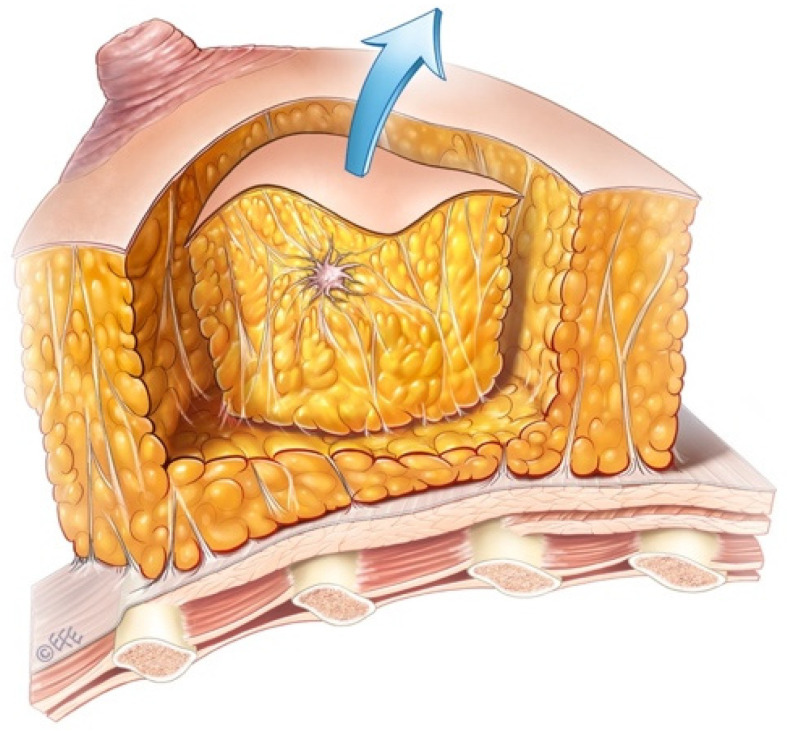
Intraoperative view of the Sparrow (Modified Lazy-S) incision. The incision follows a curved pattern with a selective central skin islet (arrow). The image is anonymized, showing no patient-identifying features.

Sparrow (Modified Lazy-S) incision is a versatile technique that can be applied to tumors in any quadrant during breast-conserving surgery. It provides a safe surgical margin, and a central skin islet is excised only when the tumor is close to the skin, resulting in minimal asymmetry and distortion. After tumor excision, the remaining tissue defect is typically closed by approximating the tissues over the pectoralis major fascia and beneath the skin. While other incisions vary according to the quadrant (radial or circular), the Sparrow (Modified Lazy-S) incision serves as an alternative to classical approaches, suitable for different quadrants, breast sizes, and tumor dimensions. Unlike the standard Lazy-S incision, both ends of the Sparrow incision are more curved, and a skin islet is preserved in the center of the excised tissue. It can be safely used horizontally or vertically in all four quadrants. Additionally, when indicated, the incision may include a large anterior skin islet, particularly for large or superficial tumors, which reduces the risk of anterior margin positivity. This approach achieves the goal of providing the most benefit with the least intervention (Figure 1). This novel technique (Sparrow), developed by our team, has not been previously reported in the literature.

The incision is closed following its natural curvature rather than in a straight line, which distributes tension across multiple vectors. Compared with standard linear incision or simple elliptical excision, it provides better mobilization and control of breast tissue, reduces distortion, and maintains oncological safety. This design is well tolerated during adjuvant radiotherapy and does not interfere with treatment planning. The Sparrow incision also provides adequate exposure of the glandular breast tissue, facilitating safe tumor excision and enabling placement of drains if required, ensuring proper postoperative drainage of the operative site.

#### Patient-Reported Outcomes

The Breast-Q™ questionnaires were administered once postoperatively, after completion of all adjuvant treatments, including radiotherapy, to assess the patients’ final cosmetic and psychosocial outcomes [13]. We acknowledge that including a comparison group undergoing lumpectomy through a standard incision would have strengthened the study; however, as this was a retrospective, single-arm analysis, no such control group was available.

**Figure 2:**
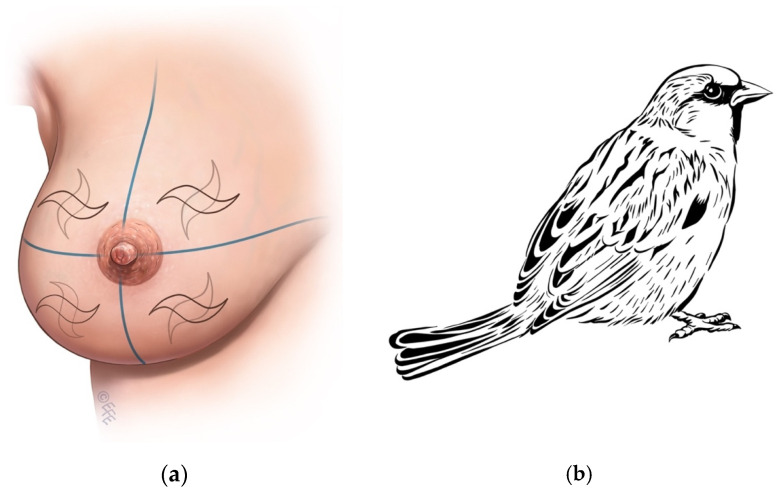
(**a**) Diagram of the Sparrow incision resembling a sparrow in shape. The skin islet location and curvature are illustrated, (**b**) Schematic showing the incision layout across different breast quadrants. Tumor position and skin islet excision are indicated.

With the long axis of the tumor forming the main body of the sparrow, Sparrow incision can be applied in all quadrants in all directions, 360 degrees. When a skin islet excision is required, its direction and length are determined according to the tumor’s extension and longitudinal diameter. It is a technique that can be used in breast-conserving surgery. It is named “Sparrow” (Figure 2a) because it resembles a sparrow in appearance (Figure 2b).

### 2.3. Statistical Analysis

SPSS 21 was used to conduct statistical analyses. Qualitative data were given as numbers (n) and percentages (%), while quantitative data were presented as mean ± SD.

## 3. Results

**Figure 3:**
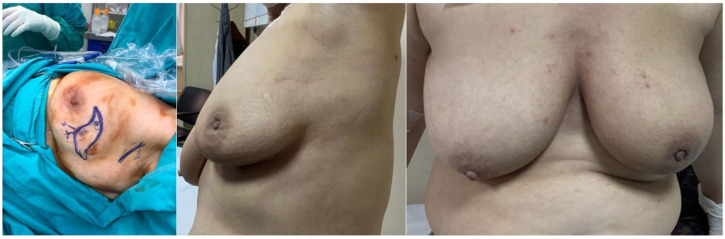
Upper outer quadrant: Intraoperative view (**left**) and 6-month postoperative follow-up (**right**) after Sparrow (Modified Lazy-S) incision. The images are anonymized to protect patient identity, showing preserved breast contour and minimal scarring.

**Figure 4:**
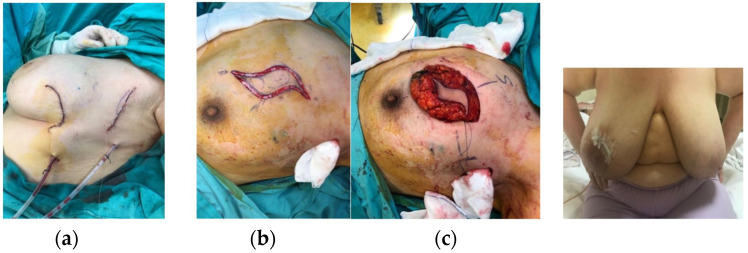
Application of the Sparrow (Modified Lazy-S) incision in different quadrants: (**a**) Lower outer quadrant, early postoperative view; (**b**) Upper inner quadrant, perioperative view; (**c**) Upper inner quadrant, early postoperative view.

Between January 2017 and January 2020, 57 patients who underwent breast-conserving surgery using the Sparrow (Modified Lazy-S) incision were retrospectively analyzed. The mean follow-up was 25 ± 10.06 months (range, 6–42 months). The mean age of the patients was 55.74 ± 12.18 years (range, 25–80 years). Tumors were in the right breast at 43.9% (n = 25) and in the left breast in 56.1% (n = 32). Regarding tumor quadrant, 50.9% (n = 29) were in the upper outer quadrant, 19.3% (n = 11) in the upper inner quadrant, 14.0% (n = 8) in the lower outer quadrant, and 15.8% (n = 9) in the lower inner quadrant (Figure 3 and Figure 4). The mean surgical margin was 0.43 ± 0.25 cm (range 0.1–0.9 cm) in the remaining 32 patients. For clarity, the previously stated 0.3 cm referred specifically to a single patient with the closest anterior margin. The overall anterior margin in the cohort ranged from 0.1 to 0.9 cm.

All patients received standard surgical management, including sentinel lymph node sampling, with axillary dissection performed when clinically indicated. The majority of patients had drains placed postoperatively, and the overall complication rate was low. Only two patients required reoperation due to concerns regarding surgical margins, highlighting the oncological safety of the Sparrow incision.

Comorbidities and demographic characteristics varied among patients, yet the technique was successfully applied across different breast volumes and tumor locations, including patients with ptotic or small breasts. Neoadjuvant therapy was administered selectively to patients with larger tumors to facilitate optimal breast conservation.

Overall, the Sparrow (Modified Lazy-S) incision was associated with low complication rates, effective tumor excision, and broad applicability in diverse patient populations. A detailed summary of patient demographics, tumor characteristics, comorbidities, and postoperative complications is presented in Table 1.

## 4. Discussion

**Figure 5:**
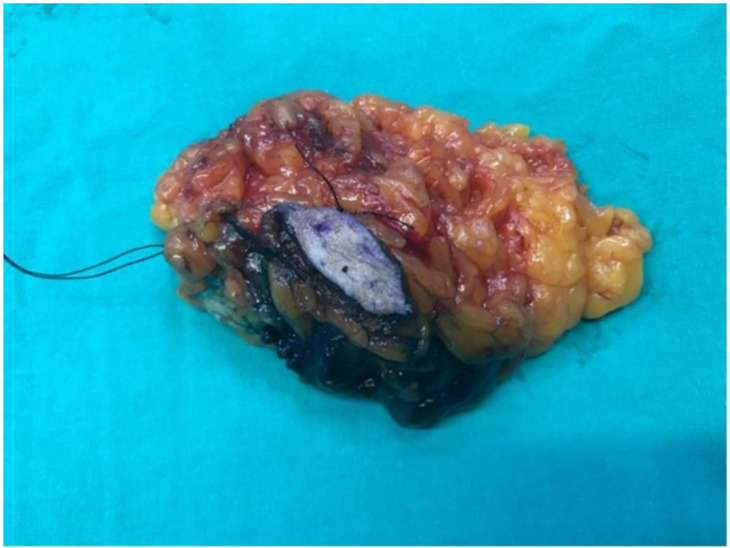
Postoperative result after tumor excision with the Sparrow incision. Tension vectors are distributed across multiple directions, minimizing contour distortion.

**Figure 6:**
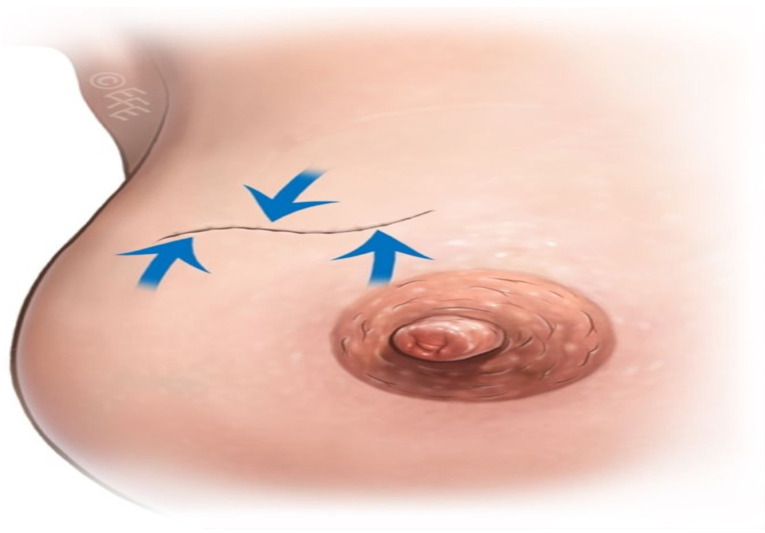
Illustration of tension vector distribution achieved by the Sparrow incision compared with a standard linear incision.

With the advances in imaging, screening programs, and patient awareness, early and rapid diagnosis and treatment of breast cancer can be achieved [14], increasing the feasibility of breast-conserving surgery. The Sparrow (Modified Lazy-S) incision, developed from the classical Lazy-S design, provides an oncologically safe approach, particularly for tumors located near the skin. When indicated, inclusion of a selective anterior skin islet allows for safe margin control while improving esthetic outcomes by reducing wound tension and distortion (Figure 5 and Figure 6).

In small breasts or cases with large tumors, extensive glandular flaps or level I reconstruction may cause displacement of the tumor bed and increase the distance between the skin incision and the original tumor site, creating challenges for adjuvant radiotherapy [15,16]. This can complicate radiotherapy planning, increase the required boost dose, and inadvertently irradiate healthy tissue, potentially affecting cosmetic outcomes [15,17,18]. The Sparrow (Modified Lazy-S) incision, closed along its natural curvature, distributes tension across multiple vectors, allowing for better mobilization and control of breast tissue, reducing distortion, and maintaining oncological safety. Because the incision preserves the tumor’s original location, it facilitates adjuvant treatment and radiotherapy planning. The Sparrow incision may offer potential advantages, as it does not require specialized instruments, prostheses, or additional operative time [19,20]. While a formal economic analysis was not performed in this retrospective study, future prospective studies could objectively assess its financial advantages compared with standard incisions.

The inclusion of a skin islet is performed selectively based on the tumor’s proximity to the skin. In smaller-breasted women, especially when the tumor is near the skin, this approach can be safely applied, minimizing esthetic deformity while maintaining oncological safety. The Sparrow incision was used in small breasts, large tumors, and tumors close to the anterior margin, with skin islets excised according to the tumor’s size and location. For larger tumors, excision included the posterior fascia. In our series, the largest anterior skin islet measured approximately 4 × 3 cm, and cosmetic outcomes remained acceptable due to the incision’s design, which distributes tension vectors and minimizes breast contour distortion.

**Figure 7:**
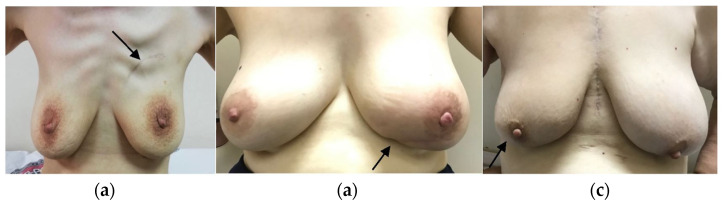
One-year postoperative follow-up of the Sparrow (Modified Lazy-S) incision in different quadrants: (**a**) Left breast, upper inner quadrant; (**b**) Left breast, lower inner quadrant; (**c**) Right breast, lower outer quadrant.

Breast cancers most commonly occur in the upper outer quadrant (45%), followed by the upper inner (15%), lower outer (10%), and lower inner quadrants (5%) [5]. In our study, tumors were located in the upper outer quadrant in 50.9% (n = 29), upper inner in 19.3% (n = 11), lower outer in 14% (n = 8), and lower inner in 15.8% (n = 9), consistent with the literature. The Sparrow incision can be applied to all quadrants (Figure 7a–c).

In a meta-analysis by Xiaopeng Cai et al., complications in breast-conserving surgery were reported at 10.97% [21], while Serrurier et al. observed a rate of 9.8% [14]. In our series, the complication rate was 5.3%, consistent with the literature.

The Sparrow incision adheres to oncological principles and is a potential advantage, as it does not prolong operative time or require additional instruments, including prostheses. It allows for excision of the previous biopsy site and can be safely applied in patients receiving neoadjuvant therapy or with multifocal tumors. Patient-reported outcomes, assessed using the Breast-Q™ questionnaire once postoperatively after completion of all adjuvant treatments, including radiotherapy [13], demonstrated high satisfaction: psychosocial well-being was 90.33%, preoperative breast satisfaction was 86.47%, and postoperative breast satisfaction was 90.14%. These results reflect positive outcomes in psychosocial well-being, breast shape, symmetry, and contour.

### The Limitations of This Study

This study has several limitations, including its retrospective design, relatively small sample size, a lack of a comparator group (e.g., standard lumpectomy incision), limited oncological follow-up, and the inclusion of some patients with ptotic breasts, which may affect cosmetic outcomes and the generalizability of the results. Additionally, due to patient privacy and consent constraints, a complete set of preoperative, intraoperative, and postoperative photographs for each patient could not be included. However, representative images are provided (Figure 1, Figure 5, Figure 6 and Figure 7) to illustrate the surgical technique and outcomes. Future prospective studies should aim to obtain comprehensive photographic documentation with appropriate consent for all patients.

## 5. Conclusions

The Sparrow incision, a modification of the Lazy-S technique, provides oncologically safe margins and can be applied safely in both horizontal and vertical orientations across all four breast quadrants. It may offer potential advantages while also reducing operative time, hospital stay, and complications, and not requiring specialized training. Patient-reported outcomes using the Breast-Q™ modules demonstrate high levels of satisfaction. Our findings suggest that the Sparrow incision is a safe, effective, and versatile alternative to level 1 oncoplastic reconstruction in breast-conserving surgery.

## Figures and Tables

**Table 1:** Patient demographics, tumor characteristics, comorbidities, and complications (n = 57).

Variable	n (%) or Mean ± SD (Range)
**Age (years)**	55.74 ± 12.18 (25–80)
**Follow-up (months)**	25 ± 10.06 (6–42)
**Tumor side**	
Right breast	25 (43.9)
Left breast	32 (56.1)
**Tumor quadrant**	
Upper outer	29 (50.9)
Upper inner	11 (19.3)
Lower outer	8 (14.0)
Lower inner	9 (15.8)
**Tumor size (cm)**	2.01 ± 0.95 (0.6–5)
**Number of tumor foci**	
Single	52 (91.2)
Two	4 (7.0)
Three	1 (1.8)
**Neoadjuvant therapy**	6 (10.5)
**Surgical margins**	
>1 cm	25 (43.9)
Mean margin (other patients)	0.43 ± 0.25 (0.1–0.9)
**Reoperation**	2 (3.5)
**Sentinel lymph node sampling**	57 (100)
**Axillary dissection**	15 (26.3)
**Drains used**	47 (82.5)
Drain duration (days)	4.70 ± 2.32 (1–12)
**Hospital stay (days)**	3 ± 1.46 (1–8)
**Postoperative complications**	
No complications	54 (94.7)
Methylene blue injection site ecchymosis	1 (1.8)
Wound infection	1 (1.8)
Seroma	1 (1.8)
**Comorbidities**	
None	37 (64.9)
1 comorbidity	12 (21.1)
2 comorbidities	3 (5.3)
3 comorbidities	5 (8.8)
**Comorbidity types**	
Diabetes mellitus	8 (14.0)
Hypertension	8 (14.0)
Thyroid disease	6 (10.5)
Second malignancy	5 (8.8)
Hepatitis B	2 (3.5)
Behçet’s disease	1 (1.8)
COPD	1 (1.8)
Coronary artery disease	1 (1.8)
Aspirin use	1 (1.8)
**Smokers**	7 (12.3)

## Data Availability

The datasets generated and/or analyzed during the current study are available from the corresponding author upon reasonable request.
